# The Pooled Prevalence of Attributed Factors of Suicide in Iran: A Systematic Review and Meta-analysis

**DOI:** 10.34172/aim.31276

**Published:** 2025-01-01

**Authors:** Parsa Rouzrokh, Fatemeh Abbasi Feijani, Yeganeh Moshiri, Sulmaz Ghahramani, Kamran Bagheri Lankarani

**Affiliations:** ^1^Health Policy Research Center, Institute of Health, Shiraz University of Medical Sciences, Shiraz, Iran; ^2^Faculty of Psychology and Educational Sciences, Central Tehran Branch, Islamic Azad University, Tehran, Iran

**Keywords:** Iran, Risk factors, Suicide

## Abstract

**Background:**

Suicide poses a critical global public health concern, and distinguishing between suicides and suicide attempts underscores the need for targeted interventions. This investigation aimed to determine the pooled prevalence of factors contributing to suicide in Iran, including socio-economic, demographic, and geographical factors.

**Methods:**

A systematic search was conducted across Web of Science, Scopus, PubMed, SID, Magiran, Elmnet, ISC, Irandoc, and Noormags databases up to July 2023. We included primary observational studies of acceptable quality that examined the prevalence of factors contributing to suicide in Iranian regions. The findings were screened for eligibility and quality, followed by a review of selected articles, from which data were extracted and analyzed.

**Results:**

Out of 1646 initial articles, 68 were selected for review and 54 for meta-analysis. The pooled prevalence rates of contributing factors were calculated as follows: male gender (64.3, 95% CI: 62.6‒66.0%), age over 25 (57.9%, 95% CI: 51.0‒64.5%), under diploma education (73.4%, 95% CI: 62.1‒82.3%), employment issues (66.4%, 95% CI: 59.7‒72.5%), urban living (61.7%, 95% CI: 53.8‒69.1%), past medical history (8.5%, 95% CI: 4.9‒14.2%), past psychiatric history (20.7%, 95% CI: 15.5‒27.1%), past suicidal attempt (12.2%, 95% CI: 8.5‒17.0%), substance abuse history (28.4%, 95% CI: 20.1‒38.3%), spring season (29.8%, 95% CI: 26.7‒33%), and hanging method (46.1%, 95% CI: 41.6‒50.6%). Significant regional differences were observed in the prevalence of gender, age, and suicide methods between western and non-western areas.

**Conclusion:**

This study describes key factors of suicides in Iran. Despite higher rates among those over 25, many young individuals are affected. Urban living and low educational attainment are significant factors. Moreover, notable regional differences were observed in gender, age, and suicide methods between western and non-western areas. These findings highlight the need for additional research related to record-keeping challenges and can guide Iranian health policymakers in developing strategies for screening and treating vulnerable individuals.

## Introduction

 Suicide remains a critical public health concern with profound implications globally, ranking as the fourth leading cause of death among individuals aged 15 to 19 of both sexes. Most suicidal deaths occur in low- and middle-income countries (LMICs), according to the World Health Organization (WHO) data from 2019.^1,2^ In that year, roughly 760 000 suicides were recorded, corresponding to an age-standardized mortality rate of 9.0 per 100 000 individuals for both sexes on a global scale.^3^ Also, studies have revealed a concerning trend in recent decades, with an escalation in suicide rates observed in developing countries, particularly in those located in the Eastern Mediterranean region.^4,5^ The WHO highlights that earlier suicide attempts and emotional difficulties, including mental health disorders, are key contributors to the risk of suicide. Other resources identify factors such as family relationships, religious faith, and social influences as significant risk elements. This risk is further compounded by a range of multifaceted drivers, including demographic settings, low socio-economic status, substance abuse, and familial issues. In other words, the complex phenomenon of suicide is shaped by extensive clinical and psychosocial contexts, making it crucial to examine suicide within the context of each country.^6-12^

 Several studies suggest distinguishable characteristics between individuals who complete suicide and those who attempt it, challenging interventions that solely focus on suicide attempts.^13-18^ Moreover, limited research is available on suicide groups. In Iran, this issue is particularly burdensome, with a 20-year statistical analysis revealing a mortality rate of 8.14 per 100 000 people, imposing significant costs on healthcare.^19^ The haunting reality of 200 years of life lost per 100 000 individuals due to suicidal behaviors and self-inflicted violence, serves as a stark reminder of the profound impact. However, this may only represent the tip of the iceberg, as relevant statistics are underreported due to inadequacies in the registry system, ineffective surveillance, and cultural and social-induced stigma and stereotype barriers.^20,21^

 Recent studies conducted by social science researchers reveal a concerning trend in Iran, where the suicide rate increased by 60% from 2015 to 2019, with an annual rise of 15%.^19^ Moreover, a comprehensive study that employed joinpoint regression analysis on data from 2003 to 2014 uncovered demographic changes in suicide patterns, indicating a steady increase among older men and educated women throughout the decade.^22^ This indicates a changing and intensifying trend of suicides over the past decade, reflecting shifts in the characteristics of the individuals involved. Underlying factors and the risk of suicide death diverge across different geographic regions of Iran, related to residents’ age, gender, intellectual status, social level, and education. For instance, Ilam, a western region of Iran, exhibits the highest suicide mortality rates among both males (24 per hundred thousand individuals) and females (16.2 per hundred thousand individuals), with self-immolation and hanging being more conventional methods in the west.^19,23,24^ Recognizing the considerable causes of suicide is crucial for health managers to have sufficient information for making decisions regarding effective suicide prevention strategies,^25,26^ as highlighted by several previous review studies on suicide incidence and hazards in Iran.^19,27-31^

 Assessing available articles revealed inconsistencies in the presentation and interpretation of reports. To our knowledge, no comprehensive systematic review and meta-analysis study has explored the factors contributing to the occurrence of suicide in Iran. Our systematic approach not only consolidates current knowledge but also aids in identifying gaps in the literature, guiding future research endeavors. Therefore, given that the prevalence of suicide in Iran is influenced by various factors, as mentioned above, we aimed to determine the pooled prevalence of factors contributing to suicide in Iran, including socio-economic, demographic, and geographical variables, with a particular focus on the high-rate western regions.

## Materials and Methods

###  Study Design

 In this study, we explore the prevalence of suicides in diverse geographical regions (west, non-west, and the entire country) through a comprehensive systematic review and pooled-prevalence meta-analysis. Our investigation incorporates various population characteristics, and we adhered to the Preferred Reporting Items for Systematic Reviews and Meta-Analysis (PRISMA) recommendations to ensure the rigor and transparency of our research methodology.

###  Search Strategy and Data Sources

 Until July 2023, an exhaustive systematic search was carried out across English-language materials related to our study aim “determining the pooled prevalence of factors contributing to suicide in Iran”. In the first step, a rapid search was conducted in PubMed, Scopus, and SID to ensure that no registered systematic review fully aligned with the aim of our study. No articles were found. The next part includes a comprehensive search of English databases such as Web of Science, Scopus, and PubMed, and Google Scholar, as well as Persian databases like SID, Magiran, Elmnet, ISC, Irandoc, and Noormags. No language restrictions were applied during the search. The titles, abstracts, and keywords of articles were examined using a combination of English and Persian keywords and operators, as provided in Supplementary file 1. The ‘AND’ operator was applied to link groups of words that represented distinct ideas, while the ‘OR’ operator was used to connect synonyms.

###  Study Selection

 Initially, we included all acceptable-quality primary observational studies that explored the factors contributing to the occurrence of suicide in Iranian populations. After eliminating duplicates, two independent researchers (P.R. and F.A.F.) screened the titles and abstracts of the articles to determine compatibility with the specified eligibility criteria. Subsequently, the authors reviewed the full texts of the selected articles. In cases of disagreement regarding the inclusion of a study during the screening phase, the final decision was deferred to the third author (S.G).

###  Inclusion Criteria

 The PCO components utilized in the search process were as follows: “P” (Population) denotes individuals who commit suicide, “C” (context) is Iran, and “O” (Outcome) denotes the prevalence of the suicide. Gray literature and guidelines were also reviewed. All of the articles were supposed to be peer-reviewed. The original studies included in this search are cross-sectional, case-control, cohort, or ecological.

###  Exclusion Criteria

 Articles that did not provide information about the prevalence of (completed) suicide in Iranian populations were excluded. Additionally, studies lacking socio-economic, geographic, or demographic factors were also omitted. For studies where the full text was not publicly available, requests were made to the corresponding author to obtain the full article. If no response was received after two attempts, the study was not included. Furthermore, reviews and editorials were excluded.

###  Quality Assessment

 For the comprehensive evaluation of the selected full-text studies, we employed the 22 items outlined in the STROBE checklist, focusing on study size, bias, statistical methods, and other methodological criteria. Each item was assigned one of three distinct values to quantify the article’s score: ‘0’ denoting complete absence, ‘2’ indicating complete presence, and ‘1’ representing partial presence or inapplicability. The final score for each study was computed by summing these values, leading to their categorization into three groups: 39‒44 (good - low risk of bias and compatibility with most criteria), 33‒38 (moderate - moderate risk of bias, and compatibility with some criteria), and less than 33 (poor - high risk of bias and compatibility with few described criteria).

 The selection of the final full text papers included moderate and good articles, while poor-quality ones were excluded. Similar to the screening phase, two independent authors (P.R. and F.A.F.) conducted the quality assessment process in a single-step approach with the third author (S.G.) responsible for resolving any disagreements. The overall kappa score for the ratings was also calculated to be 0.857, indicating a high level of agreement.

###  Data Extracting

 Following a thorough examination of the selected articles, two independent authors (P.R. and F.A.F.) utilized a predefined data extraction form to collect the necessary information, while any discrepancies were reviewed by the third author (S.G.). This checklist, developed in Microsoft Excel 2021, encompassed the following items: Geographical Region (west, non-west, whole country), province, first author, study duration, study type, study sample size, source of study, and the characteristics of the suicides studied, followed by the prevalence and the trend of suicides reported. The last two columns represented the quantitative and qualitative STROBE scores for each of the records.

 The mentioned characteristics comprised sex, age, education, occupation, marital status, and habitat, past medical/psychological history, past suicidal attempts, method of suicide, season, and reason of suicide. In our study, the provinces of Kurdistan, Kermanshah, Ilam, Lorestan, Hamadan, Khuzestan, and West Azerbaijan were considered part of western Iran, while the remaining provinces were classified as non-western. Studies that provided information on at least one item of the mentioned characteristics, were included in the meta-analysis.

###  Statistical Analysis

 Statistical analyses were conducted using the Comprehensive Meta-Analysis software, version 3.7 (Biostat Inc., Englewood, NJ, USA). Given the anticipated heterogeneity of true effect sizes, the random-effects model was employed. In a meta-analysis of prevalence studies, significant heterogeneity among the included studies is expected. Consequently, the I2 value may not be informative, and thus, it was omitted from this analysis.^32,33^

 Subgroup and subset analyses were performed to compare regional differences in contributing factors and to assess variations within different aspects of each factor. Publication bias was evaluated using Begg’s and Egger’s tests; a significance level of *P* < 0.05 was considered indicative of statistically significant publication bias.^34^ In cases where the results of Egger’s and Begg’s tests were inconclusive, the trim-and-fill method was employed to identify potential missing studies.^35,36^ Forest plots have been used to visually represent the pooled estimation of prevalence for each attributable factor, with the logit transformation applied to stabilize the variances of the prevalence rates before pooling.

## Results

**Figure 1:**
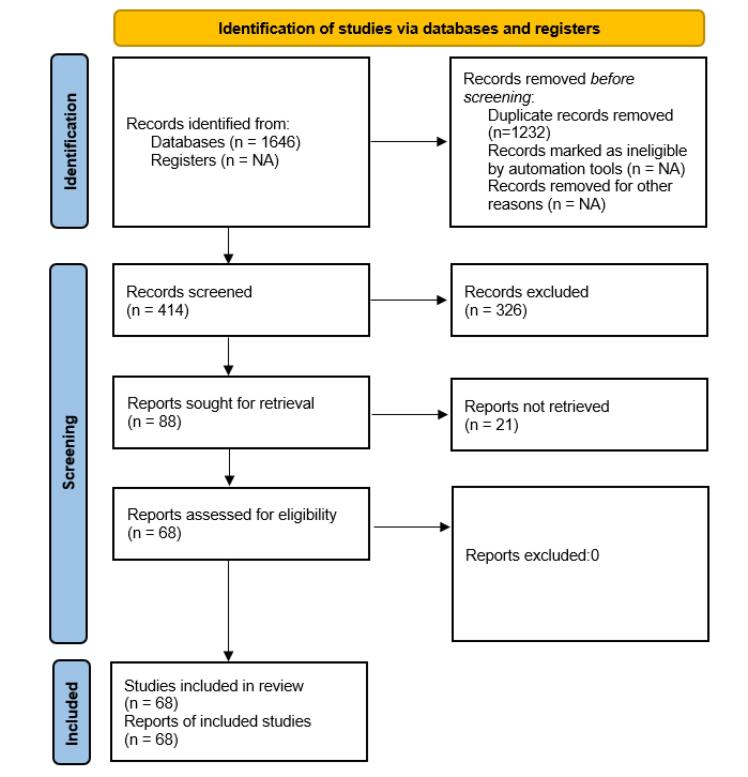
PRISMA-2020 Flow Diagram for the Article Screening Process

 A total of 1646 initial records were identified from databases, with 1,232 duplicates subsequently removed. The screening process encompassed the evaluation of titles, abstracts, and full text in the first and second phases, resulting in the selection of 68 relevant full-text studies for eligibility assessment. Guided by the STROBE checklist, all 68 articles met the eligibility criteria and were considered suitable for inclusion in the review (see Figure 1 for details).

###  Methodological Quality

 In terms of methodological quality, the STROBE checklist was employed to evaluate the records. Of these, 36 articles were deemed of moderate quality, while the remaining 32 were classified as good-quality studies. Consequently, all these studies were considered eligible for the final evaluations.

###  Description of Studies

 A comprehensive summary of the final records is presented as “Extraction Table Summary” in the Supplementary file 2.^20-22,37-102^ Among these studies, 18 focused on western regions, 32 on non-western regions, and 18 examined suicides across the entire country. These records investigated Iranian populations between 1990 and 2022, and the cross-sectional design was adopted by 51 of them.

###  Prevalence Meta-analysis

 Out of the total 68 records, only 54 contained sufficient data to proceed to the meta-analysis step. The potential contributing factors for suicides, as well as suicide methods, were quantitatively evaluated in the entire study population and among different geographical subgroups in Iran. These factors included sex (male vs. female), age (with a cut-off determined at 25 years), education (under diploma vs. diploma & higher), marital status (married vs. single/divorced/others), habitat (urban vs. rural), past medical history (with vs. without), past psychiatric history (with vs. without), substance abuse history (with vs. without), past suicidal attempts (with vs. without), and seasons (spring vs. summer vs. autumn vs. winter). Additionally, we examined the three most common methods of suicide within our study population: hanging, self-immolation, and drugs/toxins/substances.

 It is worth noting that due to limitations in the original data regarding the age of suicide victims, we only evaluated this factor using a cut-off age of 25. Furthermore, some of the articles share the same data source. Although all of these articles have been provided in the “Extraction Table”, these duplications were considered in the pooling process of our meta-analysis.

## Publication Bias and Forest Plots

**Figure 2:**
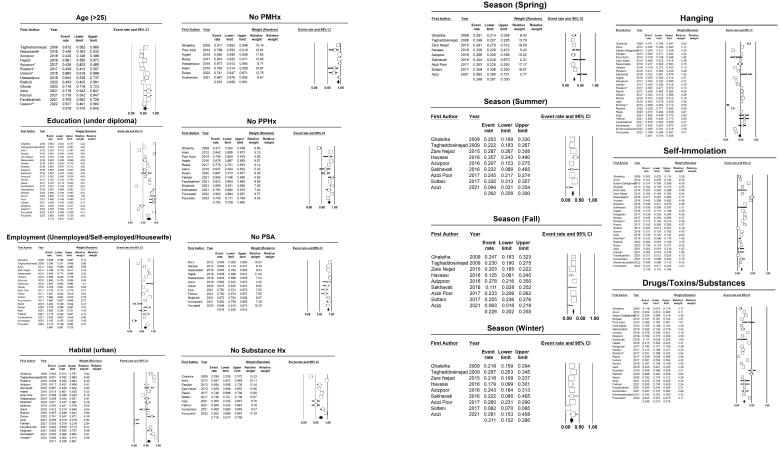
Forest Plots of the Attributable Factors of Suicide

 As detailed in the methods section, we employed Egger’s and Begg’s tests to evaluate publication biases in our analyses. The associated *P*-values are presented in Table 1. Except for education, age ( < 25 vs. > 25), and past suicidal attempts, all the values corresponding to Egger’s and Begg’s tests indicate no significant publication biases. Regarding these three factors, we applied the trim-and-fill method to identify any potential missing studies; however, no probable missing studies were identified. Moreover, the forest plots visualizing the pooled estimation of the attributable factors are presented in Figure 2.

**Table 1:** Subset Analysis (All Studies) & Publication Bias Tests Values

**Attributed Factors**	**Point Estimate (95% CI)**	**No of Studies**	**Interaction ** ***P *** **Value**	**Begg’s/Egger’s Test ** ***P *** **Value**
Sex		54	0.000	0.714/ 0.053
Male	64.3 (62.6‒66.0)			
Female	35.7 (34.0‒37.4)			
Age		14	0.002	0.352/ 0.021
> 25	57.9 (51.0-64.5)			
< 25	42.1 (35.5‒49.0)			
Education		23	0.000	0.041/ 0.243
Under Diploma	73.4 (62.1‒82.3)			
Diploma & Higher	26.6 (17.7‒37.9)			
Marital Status		33	0.096	0.136/ 0.804
Married	48.5 (45.9‒51.0)			
Single/Divorced/Others	51.5 (49.0‒54.1)			
Employment		20	0.000	0.673/ 0.411
Unemployed/Self-employed/Housewife	66.4 (59.7‒72.5)			
Others	33.6 (27.5‒40.3)			
Habitat		19	0.000	0.700/ 0.982
Urban	61.7 (53.8‒69.1)			
Rural	38.3 (30.9‒46.2)			
Past medical history (PMHx)		8	0.000	0.710/ 0.095
PMHx	8.5 (4.9‒14.2)			
No PMHx	91.5 (85.8‒95.1)			
Past psychiatric history (PPHx)		13	0.000	0.669/ 0.134
PPHx	20.7 (15.5‒27.1)			
No PPHx	79.3 (72.9‒84.5)			
Past suicidal attempt (PSA)		12	0.000	0.783/ 0.039
PSA	12.2 (8.5-17.0)			
No PSA	87.8 (83.0‒91.5)			
Substance abuse history (Substance Hx)		10	0.000	0.928/ 0.651
Substance Hx	28.4 (20.1‒38.3)			
No Substance Hx	71.6 (61.7‒79.9)			
Season		9	0.005	
Spring	29.8 (26.7‒33.0)			0.251/ 0.703
Summer	26.2 (22.6‒30.0)			1.000/ 0.126
Fall	22.8 (20.2‒25.5)			0.348/ 0.318
Winter	21.1 (15.2‒28.6)			0.175/ 0.877
Method		27	0.000	
Hanging	46.1 (41.6‒50.6)			0.851/ 0.321
Self-Immolation	13.5 (11.0‒16.4)			0.416/ 0.860
Drugs/toxins/substances	24.6 (21.5‒27.9)			0.491/ 0.690

###  Prevalence of Potential Contributing Factors in the Entire Study Population

 The prevalence of potential contributing factors in the pooled population, as presented in Table 1, reveals several notable findings. Firstly, the data indicate a significantly higher rate of suicides among men compared to women. Additionally, the majority of victims in this study were aged over 25 years. Analysis of educational levels demonstrated that a significant portion of the population had attained education below the diploma level. Marital status, however, did not show a significant association with suicides. Nevertheless, the data revealed that unemployed individuals, self-employed individuals, and housewives were more likely to commit suicide compared to other occupational groups. Notably, urbanization emerged as a contributing factor, with higher rates of suicides observed among individuals residing in urban areas. Remarkably, the analysis suggests that the majority of cases did not have any previous medical or psychiatric conditions, nor did they have a history of previous suicide attempts. Furthermore, substance abuse was not frequently reported among the cases examined. In terms of seasonal variations, the data demonstrated that suicides were more prevalent during the spring and summer months. Among the various methods studied, hanging was found to be the most common among the pooled population, followed by the use of drugs/toxins/substances, and self-immolation.

###  Prevalence of Potential Contributing Factors Among Regional Subgroups

 The prevalence of potential contributing factors among regional subgroups in Iran (western vs. non-western vs. the whole country) is presented in Table 2. The results indicate that while men in both western and non-western provinces had significantly higher rates of suicides than women, the number of female victims in western areas exceeded that in non-western regions. Moreover, studies investigated the victims’ age have shown that individuals aged over 25 in non-western areas, and under 25 in western areas, were significantly different compared to the other regional subgroups. Another noteworthy finding is related to the method of self-immolation. The results indicate that self-immolation rates in western areas were significantly higher than in non-western areas or the overall country population. None of the other potential factors showed significant differences among the regional subgroups.

**Table 2:** Subgroup Analysis (West vs. Non-West vs. Whole Country Studies)

**Attributed Factors**	**Regional Subgroup (CI 95%)**	**No of Studies**	**Interaction ** ***P*** **-value**
Sex	Male West: 59.3 (55.7‒62.8) Non-West: 68.3 (64.0‒72.2) Total: 66.2 (63.5‒68.7)	54	0.001
	Female West: 40.7 (37.2‒44.3) Non-West: 31.7 (27.8‒36.0) Total: 33.8 (31.3‒36.5)		
Education	Under Diploma West: 67.5 (57.8‒75.9) Non-West: 72.9 (60.0‒82.8)	23	0.478
	Diploma & Higher West: 32.5 (24.1‒42.2) Non-West: 27.1 (17.2‒40.0)		
Age	> 25 West: 51.2 (44.2‒58.2) Non-West: 66.5 (59.9‒72.6) Total: 64.4 (47.9‒78.2)	14	0.006
	< 25 West: 48.8 (41.8‒55.8) Non-West: 33.5 (27.4‒40.1) Total: 35.6 (21.8‒52.1)		
Marital Status	Single/Divorced/Others West: 52.5 (48.8‒56.2) Non-West: 48.5 (42.8‒54.2) Total: 56.3 (49.8‒62.7)	33	0.204
	Married West: 47.5 (43.8‒51.2) Non-West: 51.5 (45.8‒57.2) Total: 43.7 (37.3‒50.2)		
Employment	Unemployed/Self-employed/Housewife West: 70.7 (63.2‒77.2) Non-West: 64.3 (53.3‒74.0)	20	0.316
	Others West: 29.3 (22.8‒36.8) Non-West: 35.7 (49.8‒62.7)		
Habitat	Urban West: 58.9 (36.0‒78.5) Non-West: 62.2 (53.2‒70.4)	19	0.790
	Rural West: 41.1 (21.5‒64.0) Non-West: 37.8 (29.6‒46.8)		
Past medical history (PMHx)	NA	NA	NA
Past suicidal attempt (PSA)	No PSA West: 87.5 (72.4‒94.9) Non-West: 85.9 (82.3‒88.8)	12	0.786
	PSA: West: 12.5 (5.1‒27.6) Non-West: 14.1 (11.2‒17.7)		
Past PSYCHIATRIC History (PPHx)	No PPHx West: 64.3 (17.6‒93.9) Non-West: 81.0 (76.4‒85.0)	13	0.433
	PPHx West: 35.7 (6.1‒82.4) Non-West: 19.0 (15.0‒23.6)		
Substance abuse history (Substance Hx)	No Substance Hx West: 66.3 (13.0‒96.3) Non-West: 73.7 (65.8‒80.3)	10	0.790
	Substance Hx West: 33.7 (3.7‒87.0) Non-West: 26.3 (19.7‒34.2)		
Season	Spring West: 30.9 (24.9‒37.7) Non-West: 29.8 (26.1‒33.7)	9	0.762
	Summer West: 24.3 (19.9‒29.4) Non-West: 28.1 (23.5‒33.3)		0.276
	Fall West: 22.4 (17.8‒27.8) Non-West: 22.6 (19.1‒26.5)		0.949
	Winter West: 25.0 (22.7‒27.5) Non-West: 18.6 (9.7-32.7)		0.333
Method	Hanging West: 45.0 (39.7‒50.5) Non-West: 48.8 (40.4‒57.4) Total: 41.5 (32.0‒51.7)	27	0.546
	Self-Immolation West: 20.8 (16.7‒25.5) Non-West: 6.7 (4.0‒11.0) Total: 18.4 (12.5‒26.2)		0.000
	Drugs/Toxins/Substances West: 23.8 (19.5‒28.6) Non-West: 24.8 (19.5‒31.0) Total: 25.7 (18.9‒33.9)		0.906

## Discussion

 The present study is a meta-analysis on the prevalence and factors influencing suicide in Iran, with particular attention to the western regions, known for their disproportionately high rates. Our findings reveal the key points through the included studies published from 2003 to 2023. The estimated pooled number of suicides in the included studies was approximately 250 980. The age group most affected was above 25 years. Although the overall number of suicide deaths was higher among men, especially non-western men in the country, it was observed that western women, particularly through self-immolation, had a higher rate of suicide compared to women from other backgrounds. Factors such as low education level, unemployment, freelancing, and housekeeping as occupations, specific suicide methods like hanging and employingdrugs/toxins/substances, urban living, and the seasons of summer and spring were identified as contributors to the suicide cases.

 This investigation primarily aimed to investigate the overall occurrence of suicide within Iran’s population and to explore variations in suicide rates between the western regions and other parts of the country. Eighteen out of the 58 studies focused on the western provinces. Additionally, the articles that investigated all the provinces of Iran predominantly highlighted the statistics of Ilam and Kermanshah provinces. Consequently, we conducted an analysis to comprehend the disparity in contributing factors that play a role in causing suicide in these areas compared to others.

 A significant disparity exists in the rate of suicides among different genders in the included studies, with men experiencing the highest rates. In general, the suicide rate among men was nearly twice as high as that among women, consistent with findings from various studies.^22,41,47,54,56,58,63,64,83,85,89,90,93-97,101^ Research conducted in Europe and America, with a specific focus on gender impact, has revealed estimated male-to-female ratios ranging from 3 to 4 times higher. There seem to be unidentified and intricate factors underlying this phenomenon. Some theories justify this by considering gendered perspectives, where harmful methods employed are often associated with masculinity. Moreover, it is possible that women’s tendencies to seek help and their readiness to discuss emotional issues contribute to earlier recognition, treatment, and timely assistance.^103-107^

 We identified variations in suicide rates based on both gender and region in Iran. Both western and non-western men showed higher rates of suicide than women, but this difference was less pronounced among the western men and women. However, in four studies conducted exclusively in western provinces (four in Ilam and one in Kermanshah), there were more instances of suicide among women than men. Previous studies have attributed the high burden of suicide among women in western provinces to several factors, including limited socio-economic independence, social coercion pressure, and increased family insecurity that women face in these provinces. Furthermore, self-immolation is prevalent among women in these areas due to cultural factors.^40,42,45,48,102^

 A study by Daliri et al found that the western provinces had the highest rates of female suicides. They attributed this to the region’s climatic conditions, characterized by mountains and cold temperatures, providing an additional layer of insight into the complex interplay of factors influencing suicide rates among women in western provinces.^31^

 In the context of suicides, the findings of 14 studies reveal a significant trend among individuals above the age of 25, constituting more than 57% of cases. These results align with various studies conducted in Iran consistently highlighting a higher prevalence of suicides among individuals above 25.^20,44,56,57,70,82,85,86^ Similar patterns are reflected in relevant suicide data and statistics from sources such as the CDC and Statists Research Department. According to the CDC, in both 2021 and 2022 in the United States, the age group with the highest number of suicide deaths was 25‒44 years.^108^ In England and Wales, the highest suicide rate in 2021 was observed among individuals aged 50 to 54 and 45 to 49 years, respectively.^109^

 While the age group of 15 to 24 years exhibited the highest proportion of attempts in Iran, European countries, and the US, the rate of suicides was higher among individuals aged above 25 years, and the average age of individuals who died by suicide in Iran was 34 years.^110^ Studies have cited various reasons for these differences. Psychological disorders, primarily major depressive disorder, and gender differences have been implicated as contributing factors for the higher prevalence of suicide attempts among individuals under 30. On the other hand, mental and physical health conditions, functional impairments, and significant life stressors have been identified as potential factors responsible for the higher rates of suicides among older individuals.^30,111-114^

 While our study demonstrated that the rates of suicides continue to be higher among individuals over 25 years old, it is crucial to acknowledge that 42.1% of the cases involved individuals under the age of 25, representing a significant proportion. According to the CDC in United States, in 2020, suicide ranked as the second leading cause of death among individuals aged 10 to 14, and the third leading cause of death among those aged 15 to 24. This concerning trend has been on the rise in recent years, emphasizing the need for proactive measures to be taken in addressing this age group to prevent further escalation.^115^

 Drawing on the impact of socio-economic status on suicide risk, it is assumed that literacy and suicide share an inverse relationship. The rise in suicide rates in the US could be linked to a higher proportion of people with a high school education or below.^116,117^ Our research aligns with this phenomenon, as approximately 73% of the studies indicate a higher occurrence of suicides among individuals with an education level below a high school diploma.^20,22,38,40,42,46,50,51,54,57,61,64,65,70,73,81,96,102^ Previous reviews conducted in Iran also acknowledge that having low educational attainment and experiencing academic failure are significant risk factors for suicide.^30,118^ In three of our included studies, we observed that individuals with lower educational levels tend to resort to more lethal methods, such as self-immolation, firearm use, and hanging.^40,50,96^ It appears that individuals with unemployment, low income, and insufficient knowledge about their physical and mental well-being following limited educational opportunities, tend to have a less favorable quality of life. However, the relationship between education, occupation, marital status, and their impact on suicide is highly intricate, and these factors may interact and offset each other—a complex interplay commonly referred to as “intersectionality”.^20,30,57,119-121^

 When examining the impact of low education levels, findings should be interpreted with caution. A study investigating the influence of the pandemic on suicide rates revealed a noteworthy shift. Since 2020, educated individuals have shown an increase in suicide rates attributed to heightened awareness and fear of the disease.^73^ Therefore, given that the majority of our studies pertain to years preceding the pandemic, it is advisable to conduct additional research in the post-pandemic era. This is particularly relevant considering the current economic challenges and the heightened prevalence of despair among the educated population. Such studies would allow for an exploration of the evolving effects of suicide rates in the wake of the pandemic.

 The analysis identified insufficient statistical evidence to substantiate a significant association between marital status and suicide, drawing on the collective evidence from the included studies. This finding is against the hypothesis that marriage is protective against suicide as found by Durkheim in 1897^122^ and as reported in more recent works. Consistent with our results, Fässberg et al^123^ reported that marital status has the most inconsistent relationship with suicide among social factors. Moreover, a study conducted in Jordan also showed no statistically significant association between marital status and suicide.^124^

 However, according to our meta-analysis, there was no significant difference between the relative frequency of married and non-married victims.^38,40,41,43,51,64,70,79,86,90,102^ While factors such as study design, sample size, and the heterogeneity of included studies may contribute to this non-significant finding, geographical and cultural factors also play a role in shaping the profile of suicide. Notably, the protective effect of marriage is culture-specific.^125^

 In our meta-analysis, we found that over 65% of the evaluated suicide cases were individuals who were unemployed, underemployed or lacked a stable source of income. These findings are consistent with conclusions drawn from multiple studies.^20,37,38,40,42,56,57,61,64,65,73,80,83,86,102^ Unemployment and inadequate employment opportunities are major formidable obstacles in Iran that predominantly affect the youth population. These difficulties can result in feelings of disillusionment and oppression, ultimately creating conditions conducive to suicide.^38,126^ It has been also reported that housewives and unemployed people in Iran may be more susceptible to suicide due to having fewer preoccupations and more idle time for overthinking about suicide plans.^65,127^

 Globally, numerous studies have highlighted the correlation between unemployment or underemployment and a higher probability of suicide. These studies have demonstrated that there is a correlation between unemployment and a higher likelihood of suicidality and suicide mortality.^128^ During periods of economic recession and high unemployment rates, both society as a whole and individuals face an elevated risk of engaging in suicidal behavior and committing suicides. Lack of employment or insufficient work opportunities often lead to financial difficulties, including debt and financial strain, which in turn contribute to an increased likelihood of suicide at an individual level.^129-134^

 The analysis of living environments revealed that, overall, residing in urban areas in Iran entails a greater likelihood of experiencing suicides.^38,43,50,51,56,59,60,64,65,70,79,84,86^ This pattern is evident in numerous studies conducted in countries such as England and America.^135,136^ The elevated levels of stress in urban settings can be attributed to the heightened rates of suicide.^84^ Nonetheless, certain studies have indicated that the absence of social support in rural communities also constitutes a risk factor.^30,137^

 Our further investigations, when assessing geographical regions, showed that the overall suicide rates among western and non-western regions in Iran do not have a clear correlation with the place of residence. Studies conducted on this matter have conflicting opinions, to the extent that contradictory findings have also been observed in other countries.^136,138^ The disparity in suicide rates based on where people live was linked to urban individuals having access to drugs and rural individuals having access to firearms in a study.^46,50,102^ However, it is crucial to consider the multilayered nature of rural and urban life and thoroughly analyze the social, physical, and residential environments involved. Moreover, factors like rural-to-urban migration and environmental changes make it difficult to accurately assess this matter.

 The findings showed that less than 10% of the people who died by suicide had a diagnosed medical condition when they took their own lives. This finding is supported by numerous research studies conducted in Iran.^38,54,70,78,84,86^ Several diseases, such as AIDS, epilepsy, cancer, spinal cord injuries, and diabetes, have been reported to be associated with a higher risk of both attempted and fatal suicides in Iran.^75,139^ While most studies indicate that these diseases pose significant risks for suicides, it is important to note that low reported medical comorbidities in our studies could be attributed to limitations within Iran’s registry systems and the existing stigma attached to these diseases.^86^

 According to various studies conducted around the world, poor physical health increases the likelihood of suicide. Many illnesses, such as brain injuries, epilepsy, and sleep problems, were linked to suicide or suicide attempts. Moreover, the risk of suicide grew significantly with the number of physical health problems. Therefore, it is essential to implement primary, secondary, and tertiary prevention measures to lower suicide rates related to physical health issues.^140-142^ However, our findings are consistent with other studies that show that less than 20% of all suicides involved physical health conditions. But this percentage rose to more than 50% for older people who died by suicide, suggesting that this age group needs more healthcare interventions to reduce the chance of suicide.^143,144^

 Our pooled-prevalence meta-analysis revealed that 20.7% of individuals who died by suicide in Iran had a diagnosed psychiatric condition, consistent with findings from previous studies.^20,37,54,56,73,78,79,81,84,86^ Globally, mental illness is widely acknowledged as the most influential risk factor for suicide, alongside recent adverse life events and a history of self-harm. Individuals with a mental disorder are nearly eight times more likely to die by suicide compared to those without, and all types of mental disorders significantly predict suicide, with estimated adjusted relative risks ranging from 4.11 for dysthymia to 7.64 for major depressive disorder.^145-147^

 In our study, we observed that only 20.7% of cases in the Iranian population had a diagnosed mental illness. However, the Centers for Disease Control and Prevention (CDC) estimates that 46% of people who die by suicide in the United States had a known mental health condition,^148,149^ and of those who died by suicide in the USA, half had been diagnosed with at least one mental health condition in the year preceding their death.^150^ Several factors contribute to this difference between Iran and the United States, such as the pervasive presence of stigma,^151^ lack of awareness,^152,153^ structural and policy-related issues,^154^ cultural barriers, and limited financial resources.^155^ It is evident that individuals in countries like Iran have low rates of seeking help from formal mental health services for suicidal ideation, fearing of stigma associated with labels such as loss of faith or madness. This stigma acts as a deterrent for seeking appropriate healthcare and social support services.^156^ In addition to the factors previously discussed, this discrepancy could also arise from challenges in accurately diagnosing mental illnesses and limitations within Iran’s registration system, particularly in conducting comprehensive psychiatric evaluations for all individuals involved.^75,86^ Therefore, policymakers should seek solutions to address these issues for the future. One such initiative is the improvement of psychological health insurance coverage in Iran to 70%, effective since October 2023, as reported by Iran’s Ministry of Health.

 Based on our meta-analysis, only 12.2% of suicide cases had a history of prior attempts, indicating that the majority resulted in fatality on their initial attempt. This aligns with numerous studies in Iran.^37,40,70,79,84,86^ There is a substantial body of research conducted in Iran, as well as in the US and European countries, that highlights the importance of previous suicide attempts as a strong risk factor for suicide.^37,57,157,158^ It has also been indicated that individuals with previous attempts may have a higher fatality rate when attempting suicide.^40,86^

 However, our analysis revealed a notable proportion of suicide victims who had never attempted suicide before their final act. While this 12.2% figure may be influenced by Iran’s registry system limitations in identifying suicide history due to privacy concerns, it is essential not only to consider individuals with prior attempts but also to address other factors influencing a person’s choice to carry out their first and tragically final act.^86^

 Our findings reveal that 28.4% of suicide cases in Iran had a documented history of alcohol, opioid, and other substance abuse, as determined through autopsy tests. This discovery is supported by numerous studies conducted in Iran.^54,61,76,80,84^ The addiction history most frequently reported in our studies included smoking, opioid use, and alcohol consumption, all of which were linked to an increased risk of suicide.^20,54^ Moreover, addiction to these substances, along with psychological problems and family disputes, was reported as the most important motivation for committing suicide in Iran.^61,159^ While all substances contribute to an increased risk of suicidal behavior, alcohol and opioids were the most commonly identified substances in suicide victims (22% and 20% respectively), surpassing rates of cocaine and marijuana.^160-164^ In the United States, according to the CDC, 20% of suicide cases were associated with opioid use, and approximately 22% of the victims had illegal blood alcohol content. Similar studies conducted in Canada also reported figures of 25% for alcohol intake and 27-50% for any substance abuse. These findings collectively help explain the 30% prevalence of substance abuse among those who died by suicide in Iran.^165-167^ Unfortunately, drawing a distinction between the histories of substance abuse and having a substance in the victim’s blood after the suicide was not completely feasible due to reporting limitations.

 Our study findings indicate that the majority of suicides occurred during the spring and summer seasons, with approximately 30% specifically in the spring. These results align with findings from previous studies conducted in Iran, providing further confirmation.^38,46,61,65,80,83,101,102,168^ Seasonality patterns have always intrigued researchers regarding their association with suicide in other countries. Extensive studies have provided evidence supporting the notion that a notable seasonal effect exists on suicide rates, with the highest risk observed during the spring season.^169,170^ Moreover, research indicates that suicide attempts are more frequent during spring and summer, exhibiting 1.2‒1.7 times higher rates compared to winter. It has been suggested that seasonal variation may play a role in the modulation of suicide behavior by endogenous and/or environmental factors such as the longer duration of sunshine during these particular seasons, as opposed to autumn and winter, based on the positive correlation between the number of sunshine hours and number of serotonin-related actions including suicide.^171-173^

 Based on our regional subgroup analysis, there is no notable difference between western and non-western areas, in terms of the seasonality effect on suicides. However, it is worth noting that the patterns may differ based on factors such as the chosen method of suicide in specific regions. For instance, reports indicate that in the eastern and southern areas of Iran, self-immolation tends to occur more frequently during the summer and winter, while hanging is more prevalent in the spring. But overall, the first two seasons of the year can be considered as the deadliest in all regions of Iran, and probably other countries.^102,174-176^

 Our investigation uncovered that hanging is the predominant approach to suicide in Iran, which aligns with prior studies done in the country.^20,22,37,38,47,50,52,54,56,57,61,62,65,70,71,80,86,90-92,96,101^ Use of drugs/toxins/substances and self-immolation were the next frequently utilized methods. The pattern of suicide methods observed in the Eastern Mediterranean Region (EMR) countries mirrored our findings.^177^

 While hanging is widely recognized as a leading fatal suicide technique across various nations, it is worth mentioning that suicide techniques in Asia, specifically in EMR countries, vary significantly from those in Western countries. Unlike Western countries, where firearms are commonly used, Asian countries more frequently witness pesticide ingestion and self-immolation as the preferred methods.^178^ In three studies, among suicide deaths that occurred through poisoning, the majority of them were especially by the use of aluminum phosphide.^80,81,83^ Although medications, opioids and pesticide ingestion are more common due to accessibility, the fatality rate is greater when aluminum phosphide is employed.^29,46,59,60,72,179-181^

 Our analysis regarding the geographical variations in Iran also highlighted significant incidents of self-immolation, particularly in the western parts of the country, although occurrences are noted in other areas as well. Research indicates that self-immolation is predominantly observed among young married women with limited education, often linked to family conflicts.^29,47,50,53,66,71,77,82,94,101,182-185^ To effectively address this issue, it is important to conduct additional studies and formulate region-specific suicide prevention strategies that consider cultural, religious, and practical elements influencing the choice of suicide method.^28^ The use of multiple suicide methods during an episode presents researchers with a major challenge that has never been studied before. Therefore, forensic medicine and hospitals need to be more vigilant in determining the cause of death and further research is needed in this area.

## Limitations and Recommendations

 Over the past two decades, research in Iran has predominantly focused on suicide attempts and their risk factors, potentially leading to differences in contributing factors between fatal and attempted suicides. Many studies analyzed fatality rates associated with suicide attempts instead of concentrating on rates of the fatal ones. The scarcity of competent cohort studies on suicides in Iran hindered a more in-depth analysis of risk factors. Consequently, this review primarily reported the contributing factors of suicides and their prevalence, facing challenges in assessing changes over time due to cumulative reporting and the lack of global reports for each year. The review also highlighted a scarcity of studies delving into the primary reasons for fatal suicides. In our research, over half of the papers on the STROBE checklist demonstrated moderate quality, which may impact the validity of our findings. Consequently, interpretation of the results requires careful consideration. Future studies should strive for higher quality and address the limitations identified in prior research to enhance reliability.

 Recommendations for further research include a crucial need to shift focus towards fatal suicides, with future longitudinal studies aiming to identify specific risk factors associated with them. Researchers are encouraged to report findings comprehensively, enabling trend analysis over time, and conducting more cohort studies to explore causal relationships within this concept. Lastly, adopting a universal approach for reporting socioeconomic status factors in future studies is crucial, given their significant role in suicides.

## Conclusion

 This study describes key contributing factors to suicides in Iran, including gender (male), age (over 25), low education attainment, unemployment, specific suicide methods (e.g. hanging), and seasonal variations (spring and summer). Despite higher suicide rates among those over 25, an increasing number of young individuals are affected, underscoring the need for future research. Notably, certain risk factors (e.g. a history of mental illness) were absent in a significant percentage of cases, highlighting the necessity for additional research to understand causative factors, potentially linked to inadequate record-keeping systems.

 The meta-analysis emphasized the region-dependent nature of suicide factors, highlighting the influence of geographical and regional variations. Future studies should utilize multivariate models to collectively analyze these factors across different regions. Divergent findings in some studies may stem from variable factors over time, emphasizing the importance of consistently identifying vulnerable groups in different periods, particularly post-pandemic and economic downturns. The study seeks to guide Iran’s health policymakers in developing or modifying policies for the screening and treatment of vulnerable individuals affected by fatal suicides.

## Supplementary Files

Supplementary file 1: The Search String Used for the Literature Search

Supplementary file 2: The Extraction Table for Final Records
