# Supplementary material for: The Pooled Prevalence of Attributed Factors of Suicide in Iran: A Systematic Review and Meta-analysis
Source: Arch Iran Med. 2025 Jan 1;28(1):44–60. doi: 10.34172/aim.31276 (PMC11862400; doi:10.34172/aim.31276)
Supplement: Supplementary file 1 — The Search String Used for the Literature Search [file aim-28-44-s001.pdf]

**The Search String Used for the Literature Search:**

(**TIAB**:(("Iran" OR "Islamic Republic") **AND** ("Suicide" OR "Suicidal" OR "Completed Suicide" OR "Fatal Suicide" OR "Fatal Attempt" OR "Suicide Attempt" OR "Self-Harm") **AND** ("Variables" OR "Risk Factors" OR "Association" OR "Gender" OR "Age" OR "Education" OR "Drug" OR "Depression" OR "Alcohol Abuse" OR "Dementia" OR "Anxiety" OR "Insomnia" OR "Cognitive Impairment" OR "Psychosis" OR "Bipolar Disorder" OR "Psychiatric Disorder" OR "Psychological Factors" OR "Psychological Distress" OR "Psychotropic Drug" OR "Personality" OR "Medical Condition" OR "Physical Disorder" OR "Marital Status" OR "Living Status" OR "Social Isolation" OR "Suicidal Ideation" OR "Previous Suicide Attempt" OR "Economic Status" OR "Stressors" OR "Bereavement" OR "Ethnicity" OR "Race" OR "Recent Hospital Admission" OR "Aftercare" OR "Discharge" OR "Resilience" OR "Medical Consultant" OR "Disability" OR "Quality of Life" OR "Tobacco" OR "Hopelessness" OR "Sense of Control" OR "Lack of Affection" OR "Meaning in Life" OR "COVID-19")))
