# Supplementary material for: The Pooled Prevalence of Attributed Factors of Suicide in Iran: A Systematic Review and Meta-analysis
Source: Arch Iran Med. 2025 Jan 1;28(1):44–60. doi: 10.34172/aim.31276 (PMC11862400; doi:10.34172/aim.31276)
Supplement: Supplementary file 2 — The Extraction Table for Final Records [file aim-28-44-s002.pdf]

ID	1st author (Publish Year / Duration of study)	Region	Study Sample Size	Sex (M: Male, F: Female)	Age	Education	Occupation	Marital Status	Habitat	History<br>SA: Substance Abuse<br>PSA: Past Suicidal Attempt<br>PMHx: Past Medical History<br>PPHx: Past Psychiatric History	Methods	Season	STROBE Quantitative / Qualitative Score
1	Amiri[37]<br>(2012 / 2008-2009)	West	457	M: 364<br>F: 93<br>M/F = 3.91	10-19: 55<br>20-29: 193<br>30-39: 88<br>40-49: 51<br>50-59: 38<br>60-69: 17<br>70-79: 11<br>80-90: 4<br>Max: 20-29<br>Min: 80-90	Illiterate: 5.83%<br>Primary school: 9.7%<br>Secondary school: 19.69%<br>High school: 39.44%<br>Academic: 25.35%	Housewife: 58<br>Jobless: 32<br>Student: 21<br>Collegian: 2<br>Soldier: 10<br>Worker: 27<br>Employee: 8<br>Self-employment: 56<br>Retired: 4<br>Farmer/Shepherd: 16	Single: 112<br>Married: 132<br>Divorce/Widow: 5		PSA:<br>25 (14.2%)<br>SA:<br>24 (16.3%)<br>PPHx: 72	Drugs: 69<br>Toxins: 41<br>Opium: 5<br>Chemical: 7<br>Self-injury: 9<br>Hanging: 249 (83.6%)<br>Self-immolation: 46 (74.2%)<br>Oil/gas: 3<br>Firearm: 11 (52.4%)<br>Others: 6		41<br>(Good)
2	Ghaleiha[38]<br>(2009 / 2004-2005)	West	146	M: 128<br>F: 18<br>M/F = 7	10-20: 23 (15.8%)<br>21-30: 55 (37.7%)<br>31-40: 25 (17.1%)<br>41-50: 25 (17.1%)<br>51-60: 8 (5.5%)<br>More than 61: 10 (6.9%)	Low: 20 (13.6%)<br>Intermediate: 119 (81.7%)<br>High: 7 (4.7%)	Unemployed: 30 (20.5%)<br>Self-employed: 92 (63%)<br>Governmental: 14 (9.6%)<br>Student: 10 (6.8%)	Married: 72 (49.3%)<br>Single: 63 (43.2%)<br>Widow & Divorced: 11 (7.5%)	Urban: 101 (69.2%)<br>Rural: 45 (30.8%)	**SA:**<br>No abuse: 43 (29.5%)<br>Smoking: 25 (17.1%)<br>Opium: 14 (9.6%)<br>Alcohol & Marijuana: 8 (5.5%)<br>Heroin: 6 (1.4%)<br>Combine: 50 (34.2%)<br>**PPHx:**<br>Depression: 64 (43.8%)<br>Schizophrenia: 12 (8.2%)<br>Bipolar: 15 (10.3%)<br>No: 55 (37.7%)<br>**PMHx:**<br>Yes: 13 (8.9%)<br>No: 133 (91.1%)	Toxins: 17 (11.6%)<br>Hanging: 114 (78.1%)<br>Self-immolation: 9 (6.2%)<br>Others: 6 (4.1%)	Spring: 41 (28.1%)<br>Summer: 37 (25.3%)<br>Autumn: 36 (24.7%)<br>Winter: 32 (21.9%)	33<br>(Moderate)
3	Aziz Poor[39]<br>(2017 / 1993-2013)	West	867	M: 267<br>F: 600<br>M/F = 0.44	10-14: 37<br>15-24: 452<br>25-34: 194<br>35-44: 68<br>45-54: 50<br>55-64: 33<br>65<: 33	Illiterate: 154<br>Primary school: 171<br>Middle school: 188<br>High school & Diploma: 209<br>Higher: 47	Unemployed: 253<br>Housewife: 266<br>Self-employed: 62<br>Student: 137<br>Employee: 31<br>Farmer: 13<br>Worker: 18	Single: 464<br>Married: 386<br>Divorced or widow: 17	Urban: 336<br>Rural: 340		Self-harm: 5<br>Self-immolation: 685<br>Hanging: 126<br>Firearm: 42<br>Others: 9	Spring: 228<br>Summer: 212<br>Autumn: 202<br>Winter: 225	42<br>(Good)

ID	1st author (Publish Year / Duration of study)	Region	Study Sample Size	Sex (M: Male, F: Female)	Age	Education	Occupation	Marital Status	Habitat	History<br><br>SA: Substance Abuse<br>PSA: Past Suicidal Attempt<br>PMHx: Past Medical History<br>PPHx: Past Psychiatric History	Methods	Season	STROBE Quantitative / Qualitative Score
4	Nazarzadeh[40] (2016 / 2011-2012)	West	130	M: 61<br>F: 69<br>M/F = 0.97	<15: 4<br>15-24: 68<br>25-34: 41<br>35-44: 8<br>>45: 9	Illiterate: 16<br>Primary: 21<br>Middle: 32<br>High: 7<br>Diploma: 37<br>University: 6	Unemployed: 110<br>Employed: 20	Single: 63<br>Married: 67		PSA:<br>Yes: 19<br>No: 111	Drug: 16<br>Toxins: 10<br>Self-harm: 0<br>Hanging/Firearm: 34<br>Self-immolation: 62<br>Unknown: 8		38 (Moderate)
5	Mohamadian[41] (2019 / 2012-2016)	West	475	M: 280<br>F: 195<br>M/F = 1.43	57.9% in 21-40			Single: 233 (49.05%)<br>Married: 235 (49.47%)<br>Divorced: 6 (1.26%)<br>Widow: 3 (0.63%)<br>Unknown: 8 (1.68%)					37 (Moderate)
6	Veisani[42] (2016 / 2010-2014)	West	546	M: 255<br>F: 291<br>M/F = 1.14	<20: 9<br>20-29: 299<br>30-39: 186<br>40-49: 49<br>50-59: 45<br>>60: 28<br><br>Max: 20-29<br>Min: <20	Illiterate: 133<br>Primary school: 82<br>Guidance/high school: 157<br>Diploma: 136<br>University: 38	Housewife: 190<br>Unemployment: 186<br>Student: 42<br>Employed: 107<br>Others: 21	Married: 289<br>Single: 257	Urban: 7%<br>Rural: 10%		Drugs: 2%<br>Self-immolation: 44%<br>Toxin(chalk): 38%<br>Hanging: 31%		39 (Good)
7	Veisani[43] (2022 / 2014-2018)	West	445	M: 272<br>F: 183<br>M/F = 1.48	Max: 15-29: 206 (45.3%)	Illiterate/ Primary school: 202 (44.4%)<br>Guidance/high school: 145 (31.9%)<br>Diploma: 92 (20.2%)<br>Academic degree: 14 (3.3%)		Single: 236 (51.9%)<br>Married: 219 (48.1%)	urban: 394 (86.6%)<br>rural: 61 (13.4%)				35 (Moderate)
8	Veisani[44] (2018 / 2011-2016)	West	Total: 539<br><br>2011(112)<br>2012(91)<br>2013(85)<br>2014(104)<br>2015(82)<br>2016(65)	M: 324<br>F: 215	<15: 4<br>15-24: 179<br>25-44: 249<br>45-64: 73<br>>=65: 45								33 (Moderate)

ID	1st author (Publish Year / Duration of study)	Region	Study Sample Size	Sex (M: Male, F: Female)	Age	Education	Occupation	Marital Status	Habitat	History<br>SA: Substance Abuse<br>PSA: Past Suicidal Attempt<br>PMHx: Past Medical History<br>PPHx: Past Psychiatric History	Methods	Season	STROBE Quantitative / Qualitative Score
9	Havassi[45]<br>(2016 / 2005-2011)	West	56	M:25<br>F: 31<br>M/F = 0.8	< 20: 13<br>20-29: 20<br>30-45: 11<br>45<: 12	Illiterate: 12<br>Literate: 37	unemployed: 6<br>housewife: 4	Single: 23<br>Married: 33			Self-harm: 3<br>Self-immolation: 44<br>Drug/toxin: 5<br>Others: 4	Spring: 19<br>Summer: 20<br>Autumn: 7<br>Winter: 10	38<br>(Moderate)
10	Azizpoor[46]<br>(2016 / 1993-2013)	West	169	M: 94<br>F: 75<br>M/F = 1.25	10-14: 2<br>15-24: 96<br>25-34: 36<br>35-44: 17<br>45-54: 6<br>55-64: 4<br>> 65: 8	Illiterate: 24<br>Primary: 22<br>Middle: 43<br>High: 56<br>University: 12	Housewife: 30<br>Unemployed: 48<br>Self-employed: 15<br>Office Worker: 5<br>Farmer: 17<br>Student: 32<br>Worker: 5	Single: 101<br>Married: 64<br>Divorced & Widow: 4	Urban: 61<br>Rural: 57		**Poisoning**:<br>Toxins: 74<br>Tablet: 90<br>Narcotic Substance: 5<br>Building Material: 0	Seasons:<br>Spring: 45<br>Summer: 35<br>Autumn: 47<br>Winter: 41<br>Day time:<br>Morning: 26<br>Noon: 14<br>Evening: 47<br>Night: 16	41<br>(Good)
11	Kangavari[47]<br>(2017 / 2006-2016)	West	Total: 7004	M: 4259<br>F: 2745<br>Total:<br>M/F = 1.56	Total:<br>32.0 ± 15.4<br>(Min: 5-14, Max:15-29)						Hanging: 2475<br>Self-Immolation: 1676<br>Poisoning: 1261<br>Others: 1443	Max: July and August (9.9%)<br>Min: January (6.8%)	40<br>(Good)
12	Heidari[22]<br>(2017 / 2003-2014)	West	3105	M: 1909<br>F: 1196<br>M/F = 1.59	Male: 33.58 ± 15.88<br>Female: 30.22 ± 14.76	Illiterate/Primary: 1219 (39.3%)<br>Middle- high School: 1111 (35.8%)<br>Diploma and higher: 501 (16.2%)		Single: 1507 (48.5%)<br>Married: 1408 (45.3%)<br>Others: 167 (6.2%)			Hanging: 1243 (40%)<br>Self-immolation: 760 (24.5%)<br>Drugs/Toxins: 659 (21.3%)<br>Others: 429 (13.9%)<br>ASR of suicide per100K:<br>Hanging:2.7<br>self-imolation:2.7<br>poisoning:2.4		42<br>(Good)
13	Barkhordar[48]<br>(2009 / 2000-2006)	West	213	M: 69<br>F: 144<br>M/F = 0.47									33<br>(Moderate)
14	Rostami[49]<br>(2019 / 2006-2013)	West										Max:<br>August<br>(Summer)	40<br>(Good)

ID	1st author (Publish Year / Duration of study)	Region	Study Sample Size	Sex (M: Male, F: Female)	Age	Education	Occupation	Marital Status	Habitat	History<br><br>SA: Substance Abuse<br>PSA: Past Suicidal Attempt<br>PMHx: Past Medical History<br>PPHx: Past Psychiatric History	Methods	Season	STROBE Quantitative / Qualitative Score
15	Rostami[50]<br>(2016 / 2006-2013)	West	1901	All methods:<br>M: 1138<br>F: 763<br>M/F= 1.49	All methods:<br>10-19: 274<br>20-29: 790<br>30-39: 385<br>>40: 443	All methods:<br>Illiterate: 377<br>Primary/Middle: 928<br>High/Diploma: 510<br>University: 73		All methods:<br>Single: 928<br>Married: 851	All methods:<br>Urban: 1614<br>Rural: 287		**Mixed Methods:**<br><br>Drugs: 237 (12.4%)<br>Toxins: 123 (6.4%)<br>Hanging: 793 (41.7%)<br>Self-immolation: 487 (25.9%)<br>Firearms: 210 (11%)<br>Others: 45 (2.36%)		42<br>(Good)
16	Rashidi[51]<br>(2020 / 2012-2018)	West	205	M: 113<br>F: 92<br><br>M/F = 1.22	5-14: 24<br>15-24: 80<br>25-34: 49<br>35-44: 31<br>45-54: 11<br>55-64: 7<br>64 & higher: 3	Illiterate: 46<br>Primary school: 48<br>Middle school: 30<br>High school: 24<br>Diploma and higher: 27<br>Unknown: 30		Single: 77<br>Married: 96<br>Divorced or widow: 29<br>Not-reported: 3	Urban: 114<br>Rural: 87<br>Not-reported: 4		Drug: 66<br>Toxin: 34<br>Hanging: 47<br>Electrocution: 1<br>Self-immolation: 17<br>Falling: 0<br>Firearm: 20<br>Others: 20		39<br>(Good)
17	Rostami[52]<br>(2017 / 2011-2014)	West	427	M: 234<br>F: 193<br>M/F = 1.21	0-17: 51<br>18-24: 180<br>25-34: 117<br>>35: 79<br><br>Highest rate: 18-24<br><br>male: 10.5/100k<br>female: 9/100k						Hanging: 224<br>Self-harm: 7<br>Toxins: 33<br>Drugs: 53<br>Self-immolation: 74<br>Falling: 8<br>Others: 28<br><br>Hanging: 3.24/100k<br>Self-harm: 1/100k<br>Drug: 0.42/100k	autumn = 1/2 other seasons	34<br>(Moderate)

ID	1st author (Publish Year / Duration of study)	Region	Study Sample Size	Sex (M: Male, F: Female)	Age	Education	Occupation	Marital Status	Habitat	History<br><br>SA: Substance Abuse<br>PSA: Past Suicidal Attempt<br>PMHx: Past Medical History<br>PPHx: Past Psychiatric History	Methods	Season	STROBE Quantitative / Qualitative Score
18	Groohi[53]<br>(2006 / 2000-2001)	West	23	All attempts:<br><br>Male: 6 (15%) mortality rate: 4.4 per 100k<br><br>Female: 34 (85%) mortality rate: 16.7 per 100k<br><br>M/F = 0.17							Self-Immolation:<br><br>kerosene (95%)		42<br>(Good)
19	Raeisi[54]<br>(2017 / 2006-2015)	Non_West	343	M/F = 2.17	<20: 87 (25.4%)<br>20-30: 133 (38.8%)<br>31-40: 77 (22.4%)<br>>40: 46 (13.4%)	Illiterate: 29 (8.5%)<br>Primary: 70 (20.4%)<br>Middle: 119 (34.7%)<br>High: 45 (13.1%)<br>Diploma: 58 (16.9%)<br>University: 22 (6.4%)	Unemployed: 51 (14.3%)<br>Housewife: 91 (84.4%)<br>Self-employed: 24 (7%)<br>Student: 35 (10.2%)<br>Employee: 59 (17.2%)<br>staff: 8 (2.3%)<br>Conscript soldiers: 28 (8.2%)	Single: 174 (50.7%)<br>Married: 158 (46.1%)<br>Others: 11 (3.2%)		SA:<br>Smoking: 43 (12.5%)<br>Opioid: 10 (2.9%)<br>Alcohol: 2 (0.6%)<br>Ecstasy: 2 (0.6%)<br>Smoking+Opioid: 22 (6.4%)<br>Opioid+Alcohol or Opioid+Ecstasy: 8 (2.3%)<br>Unknown: 256 (74.6%)<br><br>PMHx:<br>16 (4.7%)<br><br>PPHx:<br>76 (22.1%)	Drugs: 7 (2%)<br>Toxins: 13 (3.8%)<br>Hanging: 184 (53.6%)<br>Self-immolation: 83 (24.2%)<br>Falling: 7 (2%)<br>Firearms: 37 (10.8%)<br>Others: 12 (3.5%)		33<br>(Moderate)
20	Fakhari[55]<br>(2022 / 2016-2018)	Non_West	32	M: 23<br>F: 9<br>M/F = 2.57	10-25: 9 (28.1%)<br>26-40: 18 (56.2%)<br>>40: 5 (15.6%)	Primary: 10 (31.2%)<br>Middle: 19 (59.3%)<br>High school and higher: 3 (9.37%)	student: 7 (21.8%)<br>Housewife: 5 (15.6%)<br>Unemployed or Self-employed: 18 (56.2%)<br>Farmer: 2 (6.2%)	Single: 10 (31.2%)<br>Married: 21 (65.6%)<br>Widowed & Divorced: 1 (3.1%)	Urban: 2 (6.2%)<br>Rural: 30 (93.7%)				44<br>(Good)

ID	1st author (Publish Year / Duration of study)	Region	Study Sample Size	Sex (M: Male, F: Female)	Age	Education	Occupation	Marital Status	Habitat	History<br><br>SA: Substance Abuse<br>PSA: Past Suicidal Attempt<br>PMHx: Past Medical History<br>PPHx: Past Psychiatric History	Methods	Season	STROBE Quantitative / Qualitative Score
21	Fakhari[20]<br>(2021 / 2015-2018)	Non_West	32	M: 23<br>F: 9<br>M/F = 2.55	10-25: 9 (28.1%)<br>26-40: 18 (56.2%)<br>>40: 5 (15.6%)	Primary: 10 (31.2%)<br>Middle: 19 (59.3%)<br>High school and higher: 3 (9.37%)	student: 7 (21.8%)<br>Housewife: 5 (15.6%)<br>Unemployed or Self-employed: 18 (56.2%)<br>Farmer: 2 (6.2%)	Single: 10 (31.2%)<br>Married: 21 (65.6%)<br>Widowed & Divorced: 1 (3.1%)	Urban: 2 (6.2%)<br>Rural: 30 (93.7%)	SA:<br>Alcohol Abuse: 11 (34.3%)<br>Smoker: 3 (9.3%)<br>Other Substances Abuse: 2 (6.25%)<br><br>PPHx: 3<br><br>PSA:<br>Yes: 8 (25%)<br>No: 24 (75%)	Drugs/Toxins: 8 (25%)<br>Hanging: 20 (62.5%)<br>Self-immolation: 2 (6.25%)<br>Self-harm: 2 (6.25%)	Spring > Others	44 (Good)
22	Farahbakhsh[56]<br>(2021 / 2007-2018)	Non_West	1783	M: 1204 (67.5%)<br>F: 579 (32.5%)<br>M/F = 2.07	Mean ± SD: 34.07 ± 15.68<br><br>Max: 25–59: 1102 (61.82%)<br>Min: <15: 59 (3.33%)<br><br><15: 59 (3.33%)<br>15-24: 467 (26.21%)<br>25-29: 1102 (61.82%)<br>>60: 144 (8.1%)		Self-employment/ unemployment: 651 (36.5%)<br>Student (school-university): 225 (12.6%)<br>Housewife: 464 (26%)<br>Farming or farming related: 269 (15.1%)<br>Employment: 55 (3.1%)<br>Others: 119 (6.7%)	Single: 575 (32.25%)<br>Married: 1152 (64.63%)<br>Widow/Divorced: 56 (3.12%)	Urban: 1234 (69.2%)<br>Rural: 549 (30.8%)	PSA: 111 (6.2%)<br>PPHx: 316 (17.7%)	Hanging: 1098 (61.6%)<br>Drugs/Toxins: 471 (26.4%)<br>Self-injury: 125 (7%)<br>Falling: 39 (2.2%)<br>Self-immolation: 30 (1.7%)<br>Others: 14 (0.8%)		37 (Moderate)
23	Azizi[57]<br>(2021 / 2014-2017)	Non_West	32	M: 23<br>F: 9<br>M/F = 2.55	10–25: 9 (28.125%)<br>26–40: 18 (56.25%)<br>≥40: 5 (15.63%)	Primary school: 10 (31.25%)<br>Secondary school: 19 (59.38%)<br>High school and Academic: 3 (9.37%)	Student: 7 (21.87%)<br>Farming-related: 2 (6.25%)<br>Housewife: 5 (15.63%)<br>Unemployed or Self-employed: 18 (56.25%)	Single: 10 (31.25%)<br>Married: 21 (65.63%)<br>Widow/Divorced: 1 (13.12%)	Urban: 2 (6.25)<br>Rural: 30 (93.75%)<br>Live alone: 1 (3.12%)	PSA: 8 (25%)<br>SA: 2 (6.25%)<br>Alcohol (daily): 11 (34.37%)<br>Smoking (daily): 3 (9.37%)	Hanging: 20 (62.5%)<br>Drugs/Toxins: 8 (25%)<br>Self-injury: 2 (6.25%)<br>Self-immolation: 2 (6.25%)	Spring: 18 (56.25%)<br>Summer: 3 (9.37%)<br>Autumn: 2 (6.25%)<br>Winter: 9 (28.12%)	39 (Good)

ID	1st author (Publish Year / Duration of study)	Region	Study Sample Size	Sex (M: Male, F: Female)	Age	Education	Occupation	Marital Status	Habitat	History<br>SA: Substance Abuse<br>PSA: Past Suicidal Attempt<br>PMHx: Past Medical History<br>PPHx: Past Psychiatric History	Methods	Season	STROBE Quantitative / Qualitative Score
24	Najafi[58] (2013 / 2004-2009)	Non_West	1047	M: 679<br>F:363<br>M/F = 1.88									38 (Moderate)
25	Mokhtari[59] (2019 / 2011-2016)	Non_West	63	M: 47<br>F: 16<br>M/F = 2.93	> 65 Y				Urban: 36 (64.3%)<br>Rural: 20 (35.7%)		Drugs: 4<br>Toxins: 17<br>Opioid: 5<br>Hanging: 0<br>Detergent: 6<br>Firearm: 0<br>Cold-weapon: 2<br>Self-immolation: 2<br>Falling: 2<br>Others: 3		39 (Good)
26	Mokhtari[60] (2019 / 2011-2016)	Non_West	275	M: 166<br>F: 109<br>M/F = 1.52	10-14: 78 (28.4%)<br>15-19: 197 (71.6%)				Urban: 143 (58.4%)<br>Rural: 102 (41.6%)				39 (Good)
27	ZareNejad[61] (2015 / 2003-2011)	Non_West	1815	M: 1361<br>F: 454<br>M/F = 3	Male: 30.80 ± 13.89<br>Female: 29.09 ± 13.37<br>10-19: 305 (16.8%)<br>20-29: 786 (43.3%)<br>30-39: 333 (18.3%)<br>>40: 342 (18.8%)<br>Missing: 49 (2.7%)	Illiterate: 177 (9.8%)<br>Primary: 411 (22.6%)<br>Middle: 587 (32.3%)<br>High: 484 (26.7%)<br>University: 90 (5%)<br>Missing: 66 (3.6%)	Student: 193 (10.6%)<br>Housewife: 349 (19.2%)<br>Employee: 47 (2.6%)<br>Self-employment: 809 (44.6%)<br>Un-employed / Retired: 216 (11.9%)<br>Military: 82 (4.5%)<br>Others: 25 (1.4%)<br>Missing: 94 (5.2%)	Single: 1076 (59.3%)<br>Married: 642 (35.4%)<br>Missing: 97 (5.3%)		PMHx/ PPHx:<br>Yes: 445 (24.5%)<br>No: 963 (53.1%)<br>Missing: 407 (22.4%)<br>SA:<br>Yes: 637 (35.1%)<br>No: 1082 (59.6%)<br>Missing: 96 (5.3%)	Hanging: 899 (49.5%)<br>Self-immolation: 298 (16.4%)<br>Drugs: 139 (7.7%)<br>Toxins: 160 (8.8%)<br>Firearms: 256 (14.1%)<br>Falling: 6 (0.3%)<br>Cold-weapon: 13 (0.7%)	Spring: 528 (29.1%)<br>Summer: 521 (28.7%)<br>Fall: 369 (20.3%)<br>Winter: 395 (21.8%)	34 (Moderate)

ID	1st author (Publish Year / Duration of study)	Region	Study Sample Size	Sex (M: Male, F: Female)	Age	Education	Occupation	Marital Status	Habitat	History<br><br>SA: Substance Abuse<br>PSA: Past Suicidal Attempt<br>PMHx: Past Medical History<br>PPHx: Past Psychiatric History	Methods	Season	STROBE Quantitative / Qualitative Score
28	Mirahmadizadeh[62] (2022 / 2011-2018)	Non_West	2384	Total:<br>Male: 1670 (70.05%)<br>Female: 714 (29.95%)<br>Male/Female: 2.33	32.73±15.65, Max: 15-29y			Married: 898 (37.66%)<br>Single: 1011 (42.40%)			Hanging: 714 (29.94%)<br>Drugs: 301 (16.81%)<br>Toxins: 274 (11.49%)<br>Firearms: 128 (5.36%)<br>Cold weapon: 18 (0.75%)<br>Self-immolation: 233 (9.77%)<br>Alcohol poisoning: 2 (0.08%)<br>Drowning: 2 (0.08%)<br>Falling: 35 (1.46%)<br>Others: 46 (1.9%)<br>Missing: 531 (22.28%)		35 (Moderate)
29	Jahromi[63] (2022 / 2021-2022)	Non_West	6	M: 4<br>F: 2<br>M/F = 2							Drug: 1 (16%)<br>Toxin: 3 (50%)<br>Hanging: 2 (34%)		38 (Moderate)
30	Delam[64] (2020 / 2012-2017)	Non_West	27	M: 18<br>F: 9<br>M/F = 2	Mean±SD = 33.49±11.49	Illiterate: 3<br>Under Diploma: 24<br>Academic: 0	Unemployed: 7<br>Housewife: 7<br>Employee: 1<br>Student: 5<br>Self-Employed: 7	Single: 13<br>Married: 14<br>Divorced: 0<br>Deceased Wife: 0	Urban: 20<br>Rural: 7	PSA:<br>Yes: 5<br>No: 22<br><br>PMHx:<br>Yes: 7<br>No: 20<br><br>PPHx:<br>Yes: 9<br>No: 18			40 (Good)
31	Sakhavati[65] (2016 / 2011-2015)	Non_West	18	M:16<br>F: 2<br>M/F = 8		Illiterate/ under diploma: 12 (66.66%)<br>Diploma: 5 (27.77%)<br>Academic degree: 1 (5.55%)	Housewife: 2 (11.11%)<br>Unemployment: 6 (33.33%)<br>Self-employment: 9 (50%)<br>Employed: 1 (5.55%)	Married: 12 (66.66%)<br>Single: 6 (33.33%)	Urban: 12 (66.66%)<br>Rural: 6 (33.33%)		Hanging: 13 (72.22%)<br>Drugs: 1 (5.55%)<br>Self-immolation: 1 (5.55%)<br>Toxins: 1 (5.55%)<br>Firearm: 2 (11.11%)	Summer: 4 (22.2%)<br>Spring: 8 (44.4%)<br>Autumn: 2 (11.1%)<br>Winter: 4 (22.2%)	33 (Moderate)
32	Mobayen[66] (2020 / 2009-2017)	Non_West	60								**All Self-Immolation**		39 (Good)

ID	1st author (Publish Year / Duration of study)	Region	Study Sample Size	Sex (M: Male, F: Female)	Age	Education	Occupation	Marital Status	Habitat	History<br>SA: Substance Abuse<br>PSA: Past Suicidal Attempt<br>PMHx: Past Medical History<br>PPHx: Past Psychiatric History	Methods	Season	STROBE Quantitative / Qualitative Score
33	Taziki[67]<br>(2006 / 2003-2004)	Non_West	171	M: 165<br>F: 6<br>M/F = 26									33<br>(Moderate)
34	Kazemi[68]<br>(2015 / 2012-2013)	Non_West	13	M: 9<br>F: 4<br>M/F = 2.25									34<br>(Moderate)
35	Bakhsha[69]<br>(2011 / 2003-2007)	Non_West	100		Male: 23.02 ± 9.63<br>Female: 25 ± 12.99								35<br>(Moderate)
36	Hassanipour[70]<br>(2019 / 2012-2016)	Non_West	87	M: 63<br>F: 24<br>M/F = 2.62	<15: 5<br>15-24: 26<br>25-34: 35<br>35-44: 10<br>>45: 11	Uneducated: 3<br>Elementary: 4<br>Middle School: 23<br>High School: 53<br>Diploma & University: 4		Single: 40 (1.9%)<br>Married: 47 (2.2%)	Urban: 46<br>Rural: 8<br>Urban Side: 33	PSA:<br>Yes: 5 (0.2%)<br>No: 82 (3.8%)<br><br>PMHx:<br>Yes: 2 (0.1%)<br>No: 85 (4%)	Drugs: 33<br>Hanging: 36<br>Cold-weapon: 6<br>Toxins: 5<br>Firearms: 5<br>Unknown: 2		36<br>(Moderate)
37	Karimi[71]<br>(2018 / 2013-2015)	Non_West	576	All methods:<br>M: 423<br>F: 153<br>M/F = 2.76	All methods:<br>10-14: 14<br>15-29: 262<br>30-34: 181<br>45-59:72<br>>60: 39<br>Max: 15-29<br>Min: 10-14			All Methods (Single/Married):<br>261 (45.3%)<br>307 (53.3%)			**Mixed Methods:**<br><br>Drugs/Toxins: 96<br><br>Hanging: 330<br><br>Self-immolation: 87<br><br>Firearm: 29<br><br>Cold weapon: 7<br><br>Falling: 13		43<br>(Good)
38	Gheshlaghi[72]<br>(2012 / 2007-2008)	Non_West	3		all attempts:<br>65y - 83y						**Poisoning in Elders:**<br>Fatal method: Mixed drugs		33<br>(Moderate)
39	Pouradeli[73]<br>(2023 / 2017-2020)	Non_West	642	Total:<br>M: 476<br>F: 166	Mean age:31.2 ± 13	**Total:**<br>Illiterate: 69<br>Under diploma: 376<br>Diploma: 137<br>Associate: 22<br>Bachelor or higher: 38	**Total:**<br>Housewife: 123<br>Unemployed: 78<br>Student: 78<br>Governmental: 47<br>Non-Governmental: 295	**Total:**<br>Single: 306<br>Married: 297<br>Divorced: 39		**Total:**<br>**PSA**:<br>Yes: 88<br>No: 483<br>Unknown: 71<br>**PPHx:**<br>Yes: 135<br>No: 404<br>Unknown: 103			39<br>(Good)

ID	1st author (Publish Year / Duration of study)	Region	Study Sample Size	Sex (M: Male, F: Female)	Age	Education	Occupation	Marital Status	Habitat	History<br><br>SA: Substance Abuse<br>PSA: Past Suicidal Attempt<br>PMHx: Past Medical History<br>PPHx: Past Psychiatric History	Methods	Season	STROBE Quantitative / Qualitative Score
40	Moqaddasi Amiri[74]<br>(2015 / 2005-2011)	Non_West	163	M: 96<br>F: 67<br>M/F = 1.43									36<br>(Moderate)
41	Alami[75]<br>(2019 / 2009-2014)	Non_West	17	M: 12<br>F: 5<br>M/F = 2.44		Non-Academic: 16<br>Academic: 1	Governmental: 8<br>Housewife: 5<br>Other: 4	Married: 13<br>Single: 4	Urban: 7<br>Rural: 10	**PPHx:**<br>Yes: 6<br>No: 11<br>**PMHx:**<br>Yes: 4<br>No: 13<br>**PSA:**<br>Yes: 2<br>No: 15	Non-Physical: 7<br>Physical: 10		40<br>(Good)
42	Ranjbar[76]<br>(2013 / 2009-2011)	Non_West	104	M: 94<br>F:10<br><br>M/F = 9.41	mean ± SD:<br>30.9 ± 12.1					PSA: 15 (14%)<br>SA: 36 (35%)<br>(Morphine 27 (75%), Ethanol 9 (25%) specially in youngers, Codeine 8 (22%)	**hanging**		35<br>(Moderate)
43	Alaghehbandan[77]<br>(2015 / 2006-2007)	Non_West	68	All attempts: Female							**Self-Immolation:**<br><br>kerosene (97.7%)		42<br>(Good)
44	Poor Azizi[78]<br>(2014 / 2008-2009)	Non_West	19	M: 15<br>F: 4<br>M/F = 3.75				Single: 11<br>Married: 8		PMHx:<br>Positive: 4<br>Negative: 15<br>PPHx:<br>Positive: 4<br>Negative: 15	Drug: 1<br>Toxin: 6<br>Hanging: 6<br>Self-immolation: 0<br>Falling: 1<br>Firearm: 3<br>Cold-weapon: 1<br>Others: 1		33<br>(Moderate)
45	Mojahedi[79]<br>(2021 / 2014-2019)	Non_West	86	M: 47<br>F: 39<br>M/F = 1.20	31.03 ± 13.51	Under-diploma: 40<br>Diploma: 19<br>University: 12<br>Other: 14		Married: 43<br>Single: 37<br>Divorced: 5	Urban: 53<br>Rural: 27<br>Rural /Urban Ratio:2	PSA: 11<br>PPHx: 9			38<br>(Moderate)

ID	1st author (Publish Year / Duration of study)	Region	Study Sample Size	Sex (M: Male, F: Female)	Age	Education	Occupation	Marital Status	Habitat	History<br><br>SA: Substance Abuse<br>PSA: Past Suicidal Attempt<br>PMHx: Past Medical History<br>PPHx: Past Psychiatric History	Methods	Season	STROBE Quantitative / Qualitative Score
46	Soltani[80]<br>(2017 / 2011-2016)	Non_West	1773	M: 1338<br>F: 435<br>Male/Female = 3.1	Total: mean (SD) = 33.19 (13.11)<br>Male: 33.52(13.05)<br>Female: 32.18(13.26)		Unemployed/ Housewife: 807(45.5%)<br>Self-employed: 725(41%)<br>Student: 147(8.3%)<br>Employee/ Retired: 72(4%)<br>Conscript soldiers: 22(1.2%)	Single: 641 (36.2%):<br>Male: 543<br>Female: 98<br><br>Married: 821 (46.3%)<br>Male:579<br>Female: 242<br><br>Separated: 227 (12.8%)<br>Male: 157<br>Female: 70<br><br>Widow: 84 (4.7%)<br>Male: 59<br>Female: 25		SA:<br>Total 482 (27.2%)<br>- hallucinogenic/ psychoactive: 352(73%)<br>- opioids: 239(50%)<br>- alcohol: 135(28%)	Hanging: 962(54.6%)<br>Poisoning: 640(35.8%)<br>Falling: 88(5%)<br>Burning: 35(2%)<br>Shooting: 29(1.6%)<br>Others: 19(1%)	Summer: 594 (33.5%)<br>Spring: 581(32.8%)<br>Autumn: 453(25.5%)<br>Winter: 145(8.2%)	35 (Moderate)
47	Forouzesh[81]<br>(2022 / 2010-2020)	Non_West	350 (age<18)	M: 167<br>F: 183<br><br>M/F = 0.91	Mean ± SD: 16.36 ± 1.76<br>Range: 11-18	Illiterate: 17 (4.9%)<br>Primary: 35 (10%)<br>Middle: 111 (31.7%)<br>High: 134 (38.3%)<br>Diploma: 22 (6.3%)<br><br>Max: High School: 134 (38.3%)<br>Min: Illiterate: 17 (4.9%)			Max: Outskirts of Tehran: 112 (32.5%)<br><br>Min: Downscales of Tehran: 94 (26.2%)	PPHx: 35<br><br>SA:<br>Addiction: 14 (4%)<br>Alcohol test in bodies: 13 (3.71%)	Hanging: 121 (34.57%)<br>Drugs/Toxins:<br>Total: 148 (42.28%):<br>Rice tablet or aluminum phosphide: 90 (25.7%)<br>Opium: 26 (7.4%)<br>TCA: 6 (1.7%)<br>Propranolol: 6 (1.7%)<br>Falling: 59 (16.85%)<br>Self-immolation: 8 (2.28%)<br>Firearms: 5 (1.42%)<br>Choking (gas): 6 (1.71%)<br>metro: 2 (0.57%)<br>Cold-weapon: 2 (0.57%)	Winter:90<br>Spring:89<br>Autumn:89<br>Summer:82	38 (Moderate)

ID	1st author (Publish Year / Duration of study)	Region	Study Sample Size	Sex (M: Male, F: Female)	Age	Education	Occupation	Marital Status	Habitat	History<br>SA: Substance Abuse<br>PSA: Past Suicidal Attempt<br>PMHx: Past Medical History<br>PPHx: Past Psychiatric History	Methods	Season	STROBE Quantitative / Qualitative Score
48	Taghaddosinejad[82] (2010 / 2001-2006)	Non_West	374	M:134(35.8%)<br>F:240(64.2%)<br>F/M = 1.8	Mean: 27.1±15.4<br>Female: 26.5±15.2<br>Male: 28.2±15.7	Illiterate: Total: 98 (26.2%)<br>Primary school: Total: 73 (19.5%)<br>Secondary school: Total: 145 (38.8%)<br>High school: Total: 54 (14.4%)<br>University: Total: 4 (1.1%)	Female: housekeeper 87.9%<br>Male: unemployed 58.2%	Single: Total: 120 (32.0%)<br>Married: Total: 228 (61.0%)<br>Engaged: Total: 19 (5.1%)<br>Widow: Total: 7 (1.9%)	suburban :251(67.1%)<br>urban:123(32.9%)		**Self-Immolation:**<br>kerosene: 271 (72.5%)<br>gasoline: 58(15.5%)<br>domestic gas: 28(7.5%)<br>alcohol: 17(4.5%)	Winter:29.9%<br>Spring:24.9%<br>Autumn:23.0%<br>Summer:22.2%	35 (Moderate)
49	Kordrostami[83] (2017 / 2011-2015)	Non_West	674	M: 462<br>F: 212<br>M/F = 2.17	Mean age :32.61 ± 13.7<br>10-15: 16<br>16-20: 109<br>21-30: 223<br>31-40: 132<br>41-50: 112<br>>50: 82	Illiterate: 19<br>Primary/Middle School: 132<br>Diploma: 357<br>University Students: 136<br>University Degree: 30	Unemployed: 186<br>Non-Governmental Employee: 152<br>Worker: 12<br>Housewife: 109<br>Governmental Employee: 46<br>Student: 136<br>Retired: 32<br>Soldier: 1	Single: 360<br>Married: 313<br>Divorced: 1			**Drugs/Toxins:**<br>Opioids: 52<br>Methamphetamine: 21<br>Ethanol: 10 (100% Male 21-40y)<br>Benzodiazepines: 6<br>TCAs: 4<br>Pesticides: 644 (Max: Aluminum phosphide)<br>Strychnine: 10<br>Poly Drugs: 50<br>Max: Opium+Ethanol+Met (22)	Spring (190 - 28.2%)> Others	40 (Good)
50	Koohestani[84] (2021 / 2018-2019)	Non_West	60	All methods:<br>M: 19<br>F: 41<br>M/F = 0.46	All methods:<br><30: 23<br>30-84: 37		Unemployed: 2 (3.6%)<br>Student: 2 (3.6%)<br>Housewife: 9 (16.4%)<br>Employed:47 (76.4%)	Single: 24 (36.8%)<br>Married: 33 (57.9%)<br>Widow: 3 (5.3%)	All methods:<br>Urban: 47<br>Rural: 13	SA:<br>All Methods:<br>Yes: 12<br>No: 48	**Mixed Methods**	**All methods (MAX): Summer**	37 (Moderate)
51	Ghodsi[85] (2020 / 1990-2015)	Whole Country	67670	M: 45,635<br>F: 22,035<br>M/F = 2.03	Max: 15-24: 18992 (28.1%)<br>Male: 29.6%<br>Female: 24.9%								43 (Good)

ID	1st author (Publish Year / Duration of study)	Region	Study Sample Size	Sex (M: Male, F: Female)	Age	Education	Occupation	Marital Status	Habitat	History<br><br>SA: Substance Abuse<br>PSA: Past Suicidal Attempt<br>PMHx: Past Medical History<br>PPHx: Past Psychiatric History	Methods	Season	STROBE Quantitative / Qualitative Score
52	Hajebi[86]<br>(2016 / 2009-2012)	Whole Country	6659	M: 3930<br>F: 2729<br>M/F = 1.43	5-14: 155 (2.3%)<br>15-25: 2765 (41.4%)<br>25-34: 1959 (29.4%)<br>35-44: 807 (12.1%)<br>45-54: 493 (7.4%)<br>55-64: 252 (3.8%)<br>>65: 226 (3.4%)<br>Missing: 11 (0.1%)		Idle: 1196 (18%)<br>Housewife: 1675 (25.2%)<br>Student: 667 (10%)<br>Self-employed: 982 (14.7%)<br>Others: 2048 (32.1%)	Married: 3173 (47.6%)<br>Single: 3022 (45.4%)<br>Widow/Widower: 45 (0.7%)<br>Divorced: 77 (1.2%)<br>Others: 342 (5.1%)	Total:<br>Urban: 4121 (61.9%)<br>Rural: 2538 (38.1%)	PSA: 429 (6.5%)<br>PPHx: 829 (12.5%)<br>PMHx: 302 (4.6%)	Drugs: 1872 (28.1%)<br>Falling: 56 (0.8%)<br>Self-immolation: 1193 (17.9%)<br>Hanging: 2018 (30.3%)<br>Cold-weapon: 55 (0.8%)<br>Firearms: 154 (2.3%)<br>Drowning: 31 (0.5%)<br>Toxins: 824 (12.4%)<br>Substance Overdose: 56 (0.8%)<br>Others: 400 (5.9%)		41<br>(Good)
53	Hajebi[87]<br>(2017 / 2014-2015)	Whole Country	1836	M: 1047<br>F: 789<br>M/F = 1.32						Item-Response Probabilities:<br>PSA: 0.549<br>PMHx: 0.253<br>PPHx: 1.010	Latent Model prevalence:<br>Without PPHx: 0.034<br>With PPHx: 0.002<br><br>Item-Response Probabilities:<br>Poisoning with substance: 0.030<br>Use of poisons: 0.238<br>Poisoning with medications: 0.269<br>Firearms: 0.094<br>Cold weapon: 0.010<br>Hanging: 0.138<br>Self-immolation: 0.729<br>Falling: 0.391<br>other ways: 0.094		38<br>(Moderate)
54	Hashemi Nazari[88]<br>(2022 / 2006-2012)	Whole Country		per 100k:<br>M: 6.75<br>F: 2.88	per 100k:<br>15-29: 9.96<br>30-49: 5.62<br>50-69: 2.96<br>70=<: 2.84								41<br>(Good)
55	Kiadaliri[89]<br>(2014 / 2006-2010)	Whole Country		M/F = 2.34									42<br>(Good)

ID	1st author (Publish Year / Duration of study)	Region	Study Sample Size	Sex (M: Male, F: Female)	Age	Education	Occupation	Marital Status	Habitat	History<br><br>SA: Substance Abuse<br>PSA: Past Suicidal Attempt<br>PMHx: Past Medical History<br>PPHx: Past Psychiatric History	Methods	Season	STROBE Quantitative / Qualitative Score
56	Izadi[90]<br>(2018 / 2006-2015)	Whole Country	35,297	M: 24736<br>F: 11191<br>M/F = 2.34	Mean ± SD:<br>Total: 32.01 ± 14.88<br>Male: 32.93 ± 15.08<br>Female: 29.85 ± 14.17			Married: 17,013 (48.95%)<br>Single: 16,425 (47.25%)<br>Divorced: 652 (1.88%)<br>Widow: 485 (1.40%)<br>Re-marriage: 184 (0.53%)			Hanging: 18,482 (52.49%)<br>Self-immolation: 4545 (12.91%)<br>Medication: 2946 (8.37%)<br>Chemical: 4984 (14.16%)<br>Firearm: 2514 (7.14%)<br>Sharp/blunt object: 275 (0.78%)<br>Drowning: 200 (0.57%)<br>Falling: 835 (2.37%)<br>Explosive: 6 (0.02%)<br>Cold weapon: 271 (0.77%)<br>Electrocution: 16 (0.05%)		43<br>(Good)
57	Razai[91]<br>(2020 / 2008-2014)	Whole Country	1601(age >60)	M: 1217<br>F: 384	Mean ± SD:<br>70.36 ± 0.17<br><br>Max: 60-74y(50.5%)<br>Min: 85<(9.9%)						Hanging: 824 (51.50%)<br>Poisoning: 308 (19.20%)<br>Self-immolation: 214 (13.40%)<br>Others/unknown: 242 (15.10%) / 13 (0.08%)		42<br>(Good)
58	Saberi-Zafaghandi[92]<br>(2012 / 2001-2007)	Whole Country	3883	M: 1893<br>F: 1913<br>M/F = 0.98	<20: 1173 (30.2%)<br>21-30: 1395 (35.9%)<br>31-40: 506 (13%)<br>41-50: 315 (8.1%)<br>>50: 332 (8.6%)<br>missing: 162 (4.2%)			Single: 1283 (33.1%)<br>Married: 1372 (35.4%)<br>Divorced: 17 (0.4%)			Drugs: 904 (23.3%)<br>Toxins: 275 (7.1%)<br>Narcotics: 40 (1%)<br>Cold-weapon: 29 (0.7%)<br>Self-immolation: 1451 (37.4%)<br>Hanging: 948 (24.4%)<br>Firearm: 96 (2.5%)<br>Others: 66 (1.7%)		39<br>(Good)

ID	1st author (Publish Year / Duration of study)	Region	Study Sample Size	Sex (M: Male, F: Female)	Age	Education	Occupation	Marital Status	Habitat	History<br><br>SA: Substance Abuse<br>PSA: Past Suicidal Attempt<br>PMHx: Past Medical History<br>PPHx: Past Psychiatric History	Methods	Season	STROBE Quantitative / Qualitative Score
59	Sharif-Alhoseini[93] (2012 / 2005-2008)	Whole Country	1200	M: 767<br>F: 433<br><br>M/F = 1.77	>13: 86<br>13-65: 1069<br>>65: 45								35 (Moderate)
60	Shojaei[94] (2012 / 2010-2011)	Whole Country	3513	M: 2477<br>F: 1036<br>M/F = 2.4									34 (Moderate)
61	Snowdon[95] (2020 / 2006-2015)	Whole Country	36034	M: 25387<br>F: 10647<br>M/F = 2.34									36 (Moderate)
62	Shojaei[96] (2014 / 2006-2010)	Whole Country	15755	All methods:<br>M: 11092<br>F: 4663<br>M/F = 2.37	(Mean age)<br>Drugs: 28.4<br>Toxins: 32.1<br>Hanging: 32.4<br>Self-immolation: 30.5<br>Firearms: 27.1<br>Others: 35.6	Illiterate:<br>Total: 2251<br>Max: Hanging (51.6%)<br>Min: Firearms (2.8%)<br><br>Primary School:<br>Total: 8018<br>Max: Hanging (56%)<br>Min: Others (3.6%)<br><br>High School:<br>Total: 4188<br>Max: Hanging (47.4%)<br>Min: Others (5.8%)<br><br>Diploma & Higher:<br>Total: 854<br>Max: Hanging (41.7%)<br>Min: Self-immolation (7.5%)					**Mixed Methods:**<br><br>Drugs: 1178 (7.4%)<br>Toxins: 1763 (11.2%)<br>Hanging: 8264 (52.2%)<br>Self-immolation: 2587 (16.4%)<br>Firearms: 1213 (7.7%)<br>Others: 746 (4.7%)		40 (Good)

ID	1st author (Publish Year / Duration of study)	Region	Study Sample Size	Sex (M: Male, F: Female)	Age	Education	Occupation	Marital Status	Habitat	History<br><br>SA: Substance Abuse<br>PSA: Past Suicidal Attempt<br>PMHx: Past Medical History<br>PPHx: Past Psychiatric History	Methods	Season	STROBE Quantitative / Qualitative Score
63	Tabesh[97]<br>(2021 / 2012-2017)	Whole Country	35093	M: 24688<br>F: 10405<br>M/F = 2.37		illiteracy rate (mean±SD): 13.35 ± 3.54	Unemployment Rate (mean ± SD): 10.97 ± 2.13<br><br>Out of Service Rate (mean±SD): 4.47±12.8	Domestic Violence Rate (mean±SD) 84.29±29.52<br><br>Divorce rate (mean±SD): 189.62±45.83	Urbanization rate (mean±SD): 68.67±12<br><br>Net Migration Rate (mean±SD): 308.14±373.97	Substance/Alcohol Abuse Rate (mean±SD): 1.66±1.62<br><br>Abortion Rate (mean±SD): 21.97 ± 5.6<br><br>Fertility Rate (mean±SD): 1.98±0.45			36 (Moderate)
64	Kazemi-Galougahi[98]<br>(2018 / 2010-2011)	Whole Country	3513										41 (Good)
65	Hajizade Asl[99]<br>(2021 / 2005-2021)	Whole Country					Unemployment Coefficient: (Based on Generalized Torque Model):<br><br>with a break period: 0.12 ± 0.01<br><br>without a break period: -0.05 ± 0.01		Urbanization Coefficient (Based on Generalized Torque Model):<br><br>With a break period: 0.06 ± 0.03<br><br>Without a break period: 0.001 ± 0.0006				34 (Moderate)
66	Sara Emamgholipour[21]<br>(2021 / 2001-2016)	Whole Country				Literacy Rate Coefficient: (Based on Panel EGLS)<br><br>Female: -1.57<br>Male: -1.99	Unemployment Rate Coefficient (Based on Panel EGLS):<br><br>Female: 0.73<br>Male: 2.67	Divorce rate: Coefficient (Based on Panel EGLS):<br><br>Female: 1.81<br>Male: 5.25	Urbanization Coefficient: (Based on Panel EGLS)<br><br>Female: 0.35<br>Male: -0.86				38 (Moderate)

ID	1st author (Publish Year / Duration of study)	Region	Study Sample Size	Sex (M: Male, F: Female)	Age	Education	Occupation	Marital Status	Habitat	History<br>SA: Substance Abuse<br>PSA: Past Suicidal Attempt<br>PMHx: Past Medical History<br>PPHx: Past Psychiatric History	Methods	Season	STROBE Quantitative / Qualitative Score
67	Hassan Haghparast-Bidgoli[100] (2018 / 2001-2010)	Whole Country		M/F = 1.03	Across all provinces (Mean ± SD):<br>15-24: 25.77% ± 1.31<br>Age<65: 5.19% ± 0.95	Across all provinces (Mean ± SD):<br>Literacy: 83.11% ± 4.24	Across all provinces (Mean ± SD):<br>Unemployment: 12.13% ± 3.72<br>2001 vs 2010 (Mean):<br>Unemployment: 15.9 vs 13	Across all provinces (Mean ± SD):<br>Divorces: 1.12 ± 0.46<br>2001 vs 2010 (Mean):<br>0.8 vs 1.6	Across all provinces (Mean ± SD):<br>Urbanization: 63.45% ± 12.07<br>2001 vs 2010 (Mean):<br>Urbanization: 60.4 vs 66.2				42 (Good)
68	Mahdavi[101] (2020 / 2016-2018)	Whole Country	9021	M: 6424<br>F: 2597<br>M/F = 2.47	Across all provinces (Mean ± SD):<br>Male: 35.8 (15.1)<br>Female: 31.5 (14.4)<br>Age group (year):<br>≤ 29: 4015 (44.6%)<br>30-59: 4328 (48.0%)<br>≥ 60:666 (7.4%)			Single: 3872 (42.9%):<br>Male: 2947<br>Female: 925<br>Married: 4742 (52.6%):<br>Male:3210<br>Female: 1532<br>others: 407 (4.5%)<br>Male: 267<br>Female: 140			Mixed Methods:<br>Hanging: 4682 (51.9%)<br>Self-poisoning: 2329 (25.8%)<br>Self-immolation: 614 (6.8%)<br>Firearms: 578 (6.4%)<br>Others: 818 (9.1%)	Spring:2426 (26.9%)<br>Summer:2471 (27.5%)<br>Fall:2026 (22.5%)<br>Winter:2079 (23.1%)	38 (Moderate)
